# Longitudinal Evolution of Bone Microarchitecture and Bone Strength in Type 2 Diabetic Postmenopausal Women With and Without History of Fragility Fractures—A 5-Year Follow-Up Study Using High Resolution Peripheral Quantitative Computed Tomography

**DOI:** 10.3389/fendo.2021.599316

**Published:** 2021-03-16

**Authors:** Ursula Heilmeier, Gabby B. Joseph, Courtney Pasco, Nhan Dinh, Soheyla Torabi, Karin Darakananda, Jiwon Youm, Julio Carballido-Gamio, Andrew J. Burghardt, Thomas M. Link, Galateia J. Kazakia

**Affiliations:** ^1^ Musculoskeletal Quantitative Imaging Research Group, Department of Radiology & Biomedical Imaging, University of California San Francisco, San Francisco, CA, United States; ^2^ Department of Rheumatology and Clinical Immunology, Faculty of Medicine, University of Freiburg, Freiburg, Germany; ^3^ Department of Bioengineering, University of California Berkeley, Berkeley, CA, United States; ^4^ Department of Radiology, University of Colorado Anschutz Medical Campus, Aurora, CO, United States

**Keywords:** diabetic bone disease, bone strength, high resolution peripheral quantitative computed tomography, bone microarchitecture, cortical porosity, secondary osteoporosis, endocortical zone, image registration

## Abstract

**Introduction:**

Diabetic bone disease is characterized by an increased fracture risk which may be partly attributed to deficits in cortical bone quality such as higher cortical porosity. However, the temporal evolution of bone microarchitecture, strength, and particularly of cortical porosity in diabetic bone disease is still unknown. Here, we aimed to prospectively characterize the 5-year changes in bone microarchitecture, strength, and cortical porosity in type 2 diabetic (T2D) postmenopausal women with (DMFx) and without history of fragility fractures (DM) and to compare those to nondiabetic fracture free controls (Co) using high resolution peripheral quantitative computed tomography (HR-pQCT).

**Methods:**

Thirty-two women underwent baseline HR-pQCT scanning of the ultradistal tibia and radius and a FU-scan 5 years later. Bone microarchitectural parameters, including cortical porosity, and bone strength estimates *via* µFEA were calculated for each timepoint and annualized. Linear regression models (adjusted for race and change in BMI) were used to compare the annualized percent changes in microarchitectural parameters between groups.

**Results:**

At baseline at the tibia, DMFx subjects exhibited the highest porosity of the three groups (66.3% greater Ct.Po, 71.9% higher Ct.Po.Volume than DM subjects, p < 0.022). Longitudinally, porosity increased significantly over time in all three groups and at similar annual rates, while DMFx exhibited the greatest annual decreases in bone strength indices (compared to DM 4.7× and 6.7× greater decreases in failure load [F] and stiffness [K], p < 0.025; compared to Co 14.1× and 22.2× greater decreases in F and K, p < 0.020).

**Conclusion:**

Our data suggest that despite different baseline levels in cortical porosity, T2D women with and without fractures experienced long-term porosity increases at a rate similar to non-diabetics. However, the annual loss in bone strength was greatest in T2D women with a history of a fragility fractures. This suggests a potentially non-linear course of cortical porosity development in T2D bone disease: major porosity may develop early in the course of disease, followed by a smaller steady annual increase in porosity which in turn can still have a detrimental effect on bone strength—depending on the amount of early cortical pre-damage.

## Introduction

Type 2 diabetes mellitus (T2D) is a worldwide growing epidemic. It is estimated that more than 350 million people suffer from T2D globally (1), and this number is projected to rise to 500 million people worldwide by 2030 (2). While certain long-term sequelae of T2D such as macro-vascular disease, retinopathy, nephropathy, and neuropathy are well recognized (3), the skeleton has only recently emerged as another important target organ subject to diabetic complications (4–6). Epidemiological studies have found that patients with T2D have an increased risk for fragility fractures despite normal or even elevated bone mineral density (BMD) (4, 5, 7). These findings suggest that diabetic bone exhibits abnormalities in bone material properties and/or microarchitecture that are independent of BMD (6). Although studies have started to investigate the factors contributing to the increased bone fragility (8–11), the mechanisms causing bone fragility in T2D remain to a large extent unclear (12–14).

High-resolution peripheral quantitative computed tomography (HR-pQCT) allows for *in vivo* visualization of bone microstructure at spatial resolutions in the order of 100 μm^3^ (15). Using this imaging modality, cross-sectional clinical studies were able to identify severe deficits in cortical bone quality, specifically increased cortical porosity, as a potential explanation for the increased prevalence of fragility fractures in T2D individuals (10, 16, 17). However, the temporal evolution of bone microarchitecture, strength, and particularly of cortical porosity in diabetic bone disease is still unknown. While several longitudinal DXA-based studies provide inconsistent data suggesting an increased rate of bone loss in T2D individuals (depending on the skeletal site) (18, 19), it remains unclear what bone compartment drives the bone loss, neither is it known how bone microarchitectural or strength parameters change over time in diabetic bone disease. Knowledge of these processes would enable the development of targeted prevention and therapies for diabetic bone disease. Thus, the aim of this study was to prospectively characterize the 5-year longitudinal changes in bone microarchitecture and strength in T2D postmenopausal women with and without history of fragility fractures and to compare their changes to non-diabetic healthy postmenopausal controls using HR-pQCT. We hypothesized that diabetic women with a history of fragility fractures will present with the largest changes in bone microarchitecture and bone strength parameters over time, in particular with the highest increase in cortical porosity at tibia and radius.

## Material and Methods

### Subjects

Thirty-two postmenopausal women were enrolled for this study. All participants had been initially seen between 2010 and 2012 as part of our baseline UCSF Diabetes Study (10, 11). Only patients that were free of any bone affecting conditions such as untreated hyperthyroidism, hyperparathyroidism, chronic renal or liver disease since their baseline study visit (20) were invited to return for this follow-up study visit. Additional exclusion criteria comprised permanent or transient immobility (all woman had to be fully mobile and walk without assistance and were not allowed to be immobilized for more than 3 months), and the chronic (>6 months) usage of bone-affecting medications (intake of estrogens, adrenal or anabolic steroids, antacids, anticoagulants, anticonvulsants, pharmacological doses of Vitamin A, fluorides, bisphosphonates, calcitonin, tamoxifen, parathyroid hormone [PTH], or thiazolidinediones). Subjects with a positive history or suspicion of bone metastasizing cancer were also excluded. In total, 12 healthy, non-diabetic controls (Co) were re-enrolled, as well as 20 diabetic subjects. Thereof, 10 were fragility-fracture free diabetics (DM), while 10 had a positive history of fragility fracture after menopause and after the onset of T2D but prior to the baseline visit (DMFx). As previously outlined, fractures of any skeletal site were eligible, but had to be caused by a low-energy trauma such as falls from standing height or less in order to qualify as a fragility fracture (10, 20). All fractures were radiographically adjudicated, and vertebral fracture status was additionally assessed in all patients *via* MRI of the thoracolumbar spine. Details of the fracture adjudication work-up can be found here (10, 20). Individuals suffering from pathologic fractures such as fractures due to a tumor-like lesion, due to local tumor growth or a focal demineralization as seen on radiographs were not eligible for the study. All subjects gave written informed consent prior to enrolment. The study was HIPAA compliant and approved by the UCSF Committee on Human Research.

### Laboratory Serum Analyses

At the baseline visit, blood samples were collected from fasting subjects as described (20). Concentrations of a standard test panel including blood glucose (mg/dl), HbA1c (%), c-peptide (ng/ml), parathyroid hormone (PTH) (pg/ml), total 25-hydroxyvitamin D (ng/ml), serum creatinine (mg/dl), serum calcium (mg/dl), were measured in a bay area branch of Quest Diagnostics. Concentrations of the bone turnover markers C-terminal telopeptide (CTX I) (ng/ml) and procollagen type 1 (P1NP) (ng/ml) were measured at Columbia University as described before (20). Based on fasting glucose and c-peptide levels, insulin resistance was then estimated by calculating the homeostasis model assessment of insulin resistance (HOMA-IR) *via* the Oxford HOMA2 online calculator version 2.2.3 (URL: http://www.dtu.ox.ac.uk/homacalculator/). This well validated and widely used computer model of the glucose-insulin feedback system computes insulin resistance (HOMA-IR) as reciprocal of insulin sensitivity (%S) according to the formula: HOMA-IR = 100/%S, with higher values suggesting insulin resistance (21, 22). To assess the baseline estimated glomerular filtration rate (eGRF), we used the Modification of Diet in Renal Disease equation (MDRD) and also corrected for race in African-American women, accordingly (23, 24). At the follow-up visit, no blood draw was performed. However, in order to still be able to collect information about kidney function and glycemic control in our patients at the time of their follow-up visit, all participants including controls were asked to bring to the follow-up visit their latest primary care laboratory reports, dating from within the last 2 months prior to the follow-up study date. These primary care laboratory reports were then used to extract follow-up levels of HbA1c (%), and of eGFR. In order to ensure accuracy and comparability of these laboratory results, clinical laboratories involved in the analysis and generation of these laboratory reports were all CLIA-certified (Clinical lab improvement amendments). In addition, all 32 study participants were asked about any known impairments in their kidney function, their levels of glycemic control, and supplemental Vitamin D intake during the follow-up period.

### Patient Baseline and Follow-Up Visit

At both visits, a trained interviewer administered a series of questionnaires to capture former and current general health status, fracture and diabetes history, as well as medication use. At the follow-up visit, special attention was given to record any changes in health, fracture, and diabetes status that had occurred after the patient’s initial enrolment. The subject’s height and weight were measured on both visits using a wall-mounted stadiometer and a calibrated balance beam scale, respectively.

### Dual-Energy X-Ray Absorptiometry (DXA)

During the baseline visit, all subjects underwent DXA scans of the proximal femur on a single DXA scanner (Lunar Prodigy, GE, Milwaukee, WI, USA). Scan quality and performance was assured and monitored according to the guidelines of the International Society for Clinical Densitometry (25). DXA images were carefully scrutinized for correct positioning, correct placement of the regions of interest (ROIs), artifacts, and pathologic findings by a board-certified musculoskeletal radiologist (TML). Regions of interests with artifacts were excluded from analysis and areal BMD of the unaffected regions was obtained. T-scores of the femoral neck and total hip were calculated by comparing individual areal BMD results to the NHANES III reference database (26). No DXA scans were obtained at the follow-up visit.

### HR-pQCT Imaging

At both visits, patients underwent high resolution peripheral quantitative computed tomography (HR-pQCT) scanning at the standard ultradistal tibial and radial scan regions (10). All scans, including the follow-up scans, were performed on the same clinical HR-pQCT system (XtremeCT, Scanco Medical AG, Brüttisellen, Switzerland), which undergoes regular and strict quality control including daily density phantom scans and weekly structural phantom scans. Potential shifts due to hardware replacement or long-term drift are immediately addressed by adjustment of the calibration equation during image reconstruction. Precision errors (RMSCV%) of our HR-pQCT system have been assessed and documented in detail previously by our group (27, 28) with density-related measures ranging <1.4 CV% _RMS_, structural parameters between 1.3 and 8.9 CV% _RMS_, and strength parameters between 1.9 and 4.3 CV% _RMS_. Tibia and radius scans were performed using the default *in-vivo* imaging protocol (imaging parameters: 60 kVp, 900 mA, 100 ms integration time) as provided by the manufacturer (15, 29). In general, the extremity of the non-dominant body side was scanned. If the patient reported a history of fracture at the non-dominant extremity, the contralateral extremity was scanned. Before scanning, the patient’s extremity was immobilized in a carbon fiber cast and mounted within the scanner gantry to limit motion. Each scan volume encompassed 9.02 mm length (110 slices) and was acquired at a fixed proximal offset from the reference line (9.5 mm proximal offset for the ultradistal radius, 22.5 mm proximal offset for the ultradistal tibia scans). For each scan, 750 projections were obtained, and the effective patient dose totaled approximately 3 μSv. Images were reconstructed to a 1536 × 1536 matrix, allowing for a final nominal resolution of 82 μm isotropic voxel size. To calculate densitometric bone parameters, image attenuation values were calibrated against the attenuation values derived from a standardized hydroxyapatite (HA) phantom. To account for potential image motion, all acquired scans were visually scored for presence and severity of motion artifacts using the Pialat artifact grading scheme (30). Scans with image quality scores of 4 or 5 were rejected from further image analysis. In total, 59 out of 64 scans fulfilled the image quality criteria and were used for image analysis.

### HR-pQCT Image Analysis

#### Image Analysis, Registration, and Parameter Calculation

To ensure spatial correspondence between baseline and follow-up images, common volumes of interest (VOIs) between baseline and follow-up scans were identified before all subsequent analyses of HR-pQCT data were performed. Cortical porosity can lead over time to a “trabecularization” of the cortex in the endocortical zone. Quantification of cortical porosity can therefore be influenced by the image registration and endocortical boundary definition method used (26). As we were interested in capturing changes occurring with T2D in the cortical bone microarchitecture over time, we used two different postprocessing algorithms to assess the common cortical VOIs. In the first method, we used the standard registration and boundary identification method commonly performed and validated in longitudinal HR-pQCT studies (31–33). In this technique, the cortical region was identified independently in both the baseline and follow-up scans, which were matched slice-wise based on total cross-sectional area. This standard analysis method (28, 34) was also used in order to assess all other trabecular and cortical parameters for each measurement site and timepoint: the density parameters total volumetric bone mineral density (Tt.BMD), trabecular volumetric BMD (Tb.BMD), cortical volumetric BMD (Ct.BMD), cortical volumetric tissue mineral density (Ct.TMD), the geometric parameters cortical thickness (Ct.Th), and cortical area (Ct.Ar), the cortical microstructure parameters cortical porosity (Ct.Po [standard]) and cortical pore volume (Ct.Po.V [standard]) and cortical pore diameter (Ct.Po.DM), the trabecular microstructure parameters trabecular number (Tb.N), trabecular thickness (Tb.Th), trabecular separation (Tb.Sp), and standard deviation of trabecular separation (Tb.Sp.SD), as well as the micro-finite-element based biomechanical parameters (see below). In the second method, a gray-level-based 3D image registration method was applied to identify the common VOI between a baseline and follow-up scan (35), which computes a rigid transformation that aligns the follow-up to the baseline scan using normalized mutual information as the optimization metric. It then uses the computed transformation and identified VOI to carry forward (“map”) baseline endosteal contours to the follow-up images (36). This baseline-mapping technique assesses bone that was regarded as cortex at baseline and measures the exact same region of bone in the follow-up image, which may have gone through endocortical trabecularization over the 4–5-year follow-up period. Cortical microstructure parameters Ct.BMD [baseline-mapped], Ct.Po [baseline-mapped], Ct.Po.V [baseline-mapped], and Ct.Po.Dm [baseline-mapped] were quantified within the common VOI identified by the baseline-mapping method.

#### µFE Analysis

In order to compute the apparent biomechanical properties under uniaxial (superior-inferior) compression, linear micro-finite-element analysis (µFEA) modeling was performed for each scan volume under 1% of strain as detailed previously (37, 38). For all simulations and all bone elements, homogeneous mechanical properties were assumed and a mesh of isotropic brick elements was generated from the binary image (39). Each element was assigned an elastic modulus of 6.829 GPa and a Poisson’s ratio of 0.3 (37, 38) and reaction forces were calculated at the proximal and distal ends of the scan region for the prescribed displacements *via* an iterative solver (Scanco FE Software, Version 1.12, Scanco Medical). To simplify computation of compartmental load distribution, cortical and trabecular bone elements were labeled as different materials with identical material properties. For each model, the parameters stiffness *K*, apparent modulus *E*, and the load fraction for the cortical compartment at the distal boundary (Ct.LF) were computed. In addition, estimated failure load *F* was calculated as previously detailed (38) (40).

### Statistical Analysis

Data were checked for normality using Q-Q-plots and Shapiro-Wilk tests. Intergroup differences for demographic variables, anthropometrics, baseline diabetes status, and bone metabolism (**Table 1**) were assessed either *via* univariate analysis of variance with subsequent *post-hoc* Tukey tests, or *via* independent t-tests or Pearson’s chi-squared tests, as appropriate. In order to evaluate the differences between baseline and follow-up HR-pQCT parameter absolute changes within each group (**Table 3** and **Supplemental Table 2**), paired t-tests were performed. To standardize follow-up times and to allow for better intergroup comparability of HR-pQCT parameter changes over time, HR-pQCT parameter were expressed as annualized percent change (apc) from baseline and were calculated according to the following formula:

$$\text{apc}=\left[\left[\left(\frac{\left(\text{FU parameter}-\text{BL parameter}\right)}{\text{time to FU in days}}\right)*365\;\text{days}\right]/\text{BL parameter}\right]*100$$

**Table 1:** Characteristics of all study participants (n = 32).

	Co (n = 12)	DM (n = 10)	DMFx (n = 10)
***Demographics and Anthropometry***			
Age [years]	58.9 ± 5.5	59.0 ± 4.1	63.0 ± 6.6
Height [m]	1.62 ± 1.8	1.60 ± 2.6	1.60 ± 2.4
Body mass index at BL [kg/m^2^]	25.6 ± 4.9	26.0 ± 2.8	29.4 ± 5.5
Δ BMI change between BL and FU [kg/m^2^]	0.9 ± 1.8	−0.2 ± 1.5	−1.5 ± 4.4
***Racial composition n*** [%]			
Caucasian	7 [58.3]	1 [10.0]	3 [30.0]
African American	0 [0.0]	3 [30.0]	2 [20.0]
Asian	3 [25.0]	6 [60.0]	4 [40.0]
Hispanic	2 [16.7]	0 [0.0]	0 [0.0]
Pacific Islander/Native Hawaiian	0. [0.0]	0 [0.0]	0 [0.0]
***Baseline diabetic and fracture status and bone metabolism***			
Duration of type 2 diabetes [years]	n.a.	6.4 ± 4.2	11.2 ± 8.0
HbA_1_c at BL [%]	5.9 ± 0.3	7.6 ± 1.4**^$^**	7.1 ± 1.6
HbA_1_c at FU [%]	5.9 ± 0.4	8.5 ± 2.7	7.4 ± 1.9
Fasting glucose [mg/dl]	92.0 ± 10.8	157.0 ± 38.9**^$^**	132.6 ± 56.9
HOMA-IR	1.3 ± 0.6	2.7 ± 1.0**^$^**	2.0 ± 1.0
DXA Total-Hip T-score	−0.7 ± 0.9	−0.2 ± 0.5	−0.6 ± 0.6
PTH [pg/ml]	41.8 ± 14.8	38.3 ± 14.7	45.1 ± 19.3
Total 25-OH Vitamin D [ng/ml]	26.2 ± 11.6	23.8 ± 13.3	37.4 ± 12.0*
P1NP [ng/ml]	57.5 ± 15.5	44.1 ± 18.3	45.9 ± 15.4
CTX [ng/ml]	520.2 ± 208.4	307.5 ± 212.2**^$^**	260.7 ± 92.5**^$^**
eGFR (estimated glomerular filtration rate) [ml/min/1.73 m^2^]	83.1 [77.2–98.0]	100.2 [84.8–115.1]	91.3 [81.1–102.4]
Time since fragility fracture until baseline enrollment [years]	n.a.	n.a.	3.2 ± 2.1
Time to FU-visit [years]	5.2 ± 0.3	4.9 ± 0.5	4.0 ± 0.8^$^ *

Co, non-diabetic postmenopausal women without history of fragility fractures at baseline. DM, type 2 diabetic (T2D) postmenopausal women without any history of fragility fracture at baseline. DMFx, T2D postmenopausal women with a positive history of fragility fracture prior to enrollment. Data are expressed as unadjusted means ± SD. Boldface indicates significant difference (p < 0.05).

BL, baseline; FU, Follow-up; Δ, absolute change in parameter; BMI, body mass index; n.a., not applicable; HbA1c, glycated hemoglobin; DXA, Dual X-ray absorptiometry; P1NP, procollagen type 1 N-terminal propeptide; CTX, C-terminal telopeptide of type 1 collagen; HOMA-IR, homeostasis model assessment of insulin resistance.

Intergroup differences were assessed using analysis of variance (ANOVA) with subsequent post-hoc Tukey tests, independent t-tests, or Pearson’s chi-squared test as appropriate. eGFR is expressed as median [25th–75th percentile].

*p < 0.05 DM vs. DMFx.

**^$^**p < 0.05 vs. Co group.

Linear regression models were used to compare baseline HR-pQCT bone parameters between groups (**Table 2** and **Supplemental Table 1**) and to compare the annualized percent changes in microarchitectural parameters between groups: all models were adjusted for race, the models using annualized percent change were additionally adjusted for change in BMI between baseline and follow-up, in order to account for the potential impact of BMI change on bone microarchitecture and bone strength (41). Given the relatively small sample size of our groups and in order to use a method with higher statistical power (42), we additionally calculated the annualized absolute change for each bone parameter and compared intergroup changes using the same linear regression model with adjustments for race and change in BMI as used for the percent change comparisons. Our primary analysis focused on the ultradistal tibial, as the tibia (unlike the radius) is a weight bearing skeletal region and has a thicker cortex where cortical changes including porosity may be better observable (17). As an exploratory analysis, we assessed bone microarchitectural parameters at the ultradistal radius and have presented these results in the supplemental material. In order to assess whether adjusting for age impacts the relationship between diabetes status and bone outcome measures, a sensitivity analysis using the same linear regression model as used above but with an additional adjustment for age was carried out. All statistical analyses were carried out using IBM SPSS^®^ Statistics 25.0 (IBM, Armonk, NY, USA) and STATA, version 16 (StataCorp LP, College Station, TX, USA). Statistical significance was defined as p < 0.05.

**Table 2:** Baseline bone microarchitectural parameters measured *via* HR-pQCT at the ultradistal tibia and given for healthy, non-diabetic postmenopausal female controls (Co), postmenopausal T2D women without history of fragility fractures (DM), and T2D postmenopausal women with a positive history of fragility fractures (DMFx).

Ultradistal Tibia	Baseline (adjusted) means ± SEM		
	Co (n = 12)	DM (n = 10)	DMFX (n = 10)
***Basic HR-pQCT measures***			
Tt.BMD [mg/cm^3^]	282.8 ± 16.5	283.0 ± 17.3	256.5 ± 16.3
Tb.BMD [mg/cm^3^]	171.1 ± 10.8	155.7 ± 11.3	151.8 ± 10.6
Ct.BMD [mg/cm^3^]	838.4 ± 26.2	887.6 ± 27.4	809.6 ± 25.8*
Ct. TMD [mg/cm^3^]	946.0 ± 16.1	967.4 ± 16.8	929.1 ± 15.9
Ct.Th [mm]	1.14 ± 0.1	1.19 ± 0.1	1.16 ± 0.1
Ct.Ar [mm^2^]	109.9 ± 5.2	112.2 ± 5.4	120.6 ± 5.1
Ct.Po.V [mm^3^]	80.8 ± 12.2	59.0 ± 12.8	101.4 ± 12.0*
Ct.Po [%]	9.08 ± 1.2	6.43 ± 1.3	10.69 ± 1.2*
Ct.Po.Dm [µm]	206.1 ± 7.6	198.6 ± 8.0	215.2 ± 7.5
Tb.N [mm^−1^]	1.76 ± 0.1	1.59 ± 0.1	1.68 ± 0.1
Tb.Th [µm]	82.9 ± 3.5	82.1 ± 3.7	75.2 ± 3.5
Tb.Sp [µm]	506.2 ± 47.1	564.3 ± 49.3	562.4 ± 46.4
Tb.Sp.SD [µm]	234.4 ± 47.0	255.9 ± 49.2	312.5 ± 46.3
***Biomechanics***			
Stiffness, K [kN/mm]	127.0 ± 7.2	127.5 ± 7.6	134.0 ± 7.1
App Modulus, E [MPa]	1525.6 ± 84.0	1564.1 ± 87.9	1427.6 ± 82.8
Estimated Failure Load, F [N]	6960.4 ± 348.7	6915.7 ± 364.8	7187.1 ± 343.5
Ct.LF_dist_ [%]	44.6 ± 3.1	48.9 ± 3.2	47.2 ± 3.0

HR-pQCT, high resolution peripheral quantitative computed tomography; BMD, bone mineral density; Tt.BMD, total bone mineral density; Tb.BMD, trabecular BMD; Ct.BMD, cortical BMD; Ct.TMD, cortical tissue mineral density; Ct.Th, cortical thickness; Ct.Ar, cortical area; Ct.Po.V, intracortical pore volume; Ct.Po, intracortical porosity; Ct.Po.Dm, mean cortical pore diameter; Tb.N, trabecular number; Tb.Th, trabecular thickness, Tb.Sp, trabecular separation; Tb.Sp.SD, standard deviation of intertrabecular distances; Ct.LF dist, distal cortical load fraction; App Modulus, apparent modulus.

Intergroup differences were assessed using linear regression models adjusted for race. Shown are race-adjusted means and standard errors (SEM). Boldface indicates significant difference (p < 0.05).

*p < 0.05 DM vs. DMFx.

^$^p < 0.05 vs. Co group.

## Results

### Subject Characteristics

Subject characteristics of all 32 participating postmenopausal women are presented in **Table 1**. Among groups, subjects were similar in age (mean age 60.2 ± 5.6 years, p = 0.250) and showed similar baseline BMI levels and racial composition (p = 0.457). At the time of the baseline visit all patients showed a normal to slightly osteopenic areal BMD at the total hip. DM subjects were diagnosed with T2D for an approximate amount of 6.4 ± 4.2 years, while DMFx patients had reportedly a diagnosis of T2D for about 11.2 ± 8.0 years (p = 0.109). At both timepoints, baseline and follow-up (FU), DM and DMFx subjects exhibited constantly elevated glycated hemoglobin levels in the range of 7.1 to 8.5% indicating a glycemic control slightly outside the suggested optimal target window for patients older than 50 years (43). Mean baseline serum PTH levels were within the reference ranges. On average, patients were followed up approximately 4.8 ± 0.8 years after their baseline visit. In detail, DMFx had the shortest follow-up time with an average of 4.0 ± 0.8 years, while controls had been seen for their follow-up appointment on average 5.2 ± 0.3 years and DM subjects 4.9 ± 0.5 years after their respective baseline visits. In total, 16 fragility fractures had been sustained in the DMFx group prior to baseline enrollment. Skeletal sites of these fractures comprised the ankle (n = 4), vertebra (n = 4), humerus (n = 1), wrist (n = 2), and metatarsals (n = 5). Most DMFx participants had suffered a single fracture, while four of the DMFx participants had suffered two fragility fractures and one DMFx participant had sustained three fragility fractures prior to baseline enrollment. With respect to incident fragility fractures, we observed that only two participants sustained incident fragility fractures during the follow-up period: one control suffered an ankle fracture, while the other participant was part of the DMFx group and sustained a tibia fracture and three rib fractures 1 and 3 years, respectively, into the FU-period. The BMI of DMFx subjects decreased during follow-up, while the BMI of controls and DM subjects remained unchanged or increased; however, all BMI changes were not significant (p > 0.05). With respect to secondary diabetic complications, 4/10 DMFx patients reported a confirmed diabetic neuropathy at enrollment, and two of these four DMFx patients suffered concomitantly from diabetic retinopathy. In the DM group, none of the subjects suffered from secondary diabetic complications such as diabetic retinopathy or neuropathy. In both diabetic groups, kidney function as measured *via* estimated glomerular filtration rate (eGFR) was similar between 80 to 110 ml/min. There was no significant change in eGFR during the follow up period (p > 0.05). Chart review showed that DMFx subjects were more likely to take vitamin D supplements in higher dosages and for longer time periods than controls and DM subjects, perhaps in response to their fracture history. This may account for the higher 25-OH vitamin D levels noted in the DMFx group.

### Comparison of Baseline HR-pQCT-Derived Bone Parameters

Baseline density and trabecular microstructural parameters: When examining baseline density and trabecular parameters, we noted that at both ultradistal scan sites total volumetric BMD, trabecular volumetric BMD, as well as trabecular bone structure was comparable between the three groups (**Table 2**, **Supplemental Table 1**).

Baseline cortical bone structure: At the ultradistal tibia, cortical volumetric BMD was similar to our original study (10), lowest in the DMFx group compared to DM without fractures (−9.6% Ct.BMD, p = 0.044), and −3.6% lower relative to controls, however, the latter difference did not reach statistical significance. Baseline cortical porosity was highest in the DMFx group: DMFx subjects had +71.9% greater Ct.Po.V (p = 0.021), and +66.3% greater Ct.Po (p = 0.020) than non-fractured DM subjects. In addition, DMFx subjects exhibited relative to both other groups larger pore diameters and lower cortical tissue mineral density (Ct.TMD), which can reflect both elevated (sub-resolution) microporosity and decreased tissue mineralization, relative to both other groups (44) (+8.3% Ct.Po.Dm *vs.* DM, p = 0.133; −4.1% Ct.TMD *vs.* DM, p = 0.103), however, these differences were not statistically significant. Trends at the ultradistal radius mirrored those at the ultradistal tibia (**Supplemental Table 1**).

Baseline bone strength: At baseline, neither of the two scan sites yielded significant differences in biomechanical parameters between the three groups.

### Longitudinal Changes in Bone Microarchitecture

Absolute changes, annualized absolute changes, and annualized percent changes in bone microarchitectural and strength parameters over time are shown for the ultradistal tibia (**Tables 3** and **4** and **Supplemental Table 4**) and for the ultradistal radius (**Supplemental Tables 2**, **3**, and **5**). At the ultradistal tibia, DMFx subjects exhibited during the whole FU-time significant losses in total BMD (−4.7%, p = 0.011), cortical BMD (−6.6%, p = 0.005), and cortical bone microstructure (+20.7% Ct.Po.V, p = 0.004; +22.7% Ct.Po, p = 0.003), including cortical TMD (−2.8% Ct.TMD, p = 0.005). Cortical pore diameter (Ct.Po.Dm), bone geometry parameters (Ct.Ar, Ct.Th), trabecular BMD, as well as trabecular bone structure were unchanged over time (p > 0.05) in DMFx subjects.

**Table 3:** Longitudinal absolute changes in HR-pQCT-derived bone microarchitectural parameters and biomechanical parameters measured at the ultradistal tibia and given for healthy, non-diabetic postmenopausal female controls (Co), T2D postmenopausal women without history of fragility fractures (DM), and T2D postmenopausal women with a positive history of fragility fractures (DMFx).

Ultradistal Tibia	Absolute Δ over 5.2 ± 0.3 y FU-time		Absolute Δ over 4.9 ± 0.5 y FU-time		Absolute Δ over 4.0 ± 0.8 y FU-time	
	Co (n = 12)		DM (n = 10)		DMFx (n = 10)	
	Means ± SEM	p	Means ± SEM	p	Means ± SEM	p
***Basic HR-pQCT measures***						
Tt.BMD [mg/cm^3^]	−10.04 ± 2.29	**0.001**	−4.71 ± 2.42	*0.084*	−11.50 ± 3.63	**0.011**
Tb.BMD [mg/cm^3^]	0.52 ± 1.24	0.684	2.56 ± 1.15	*0.054*	1.16 ± 1.45	0.446
Ct.BMD [mg/cm^3^]	−45.97 ± 9.21	**<0.001**	−33.0 ± 8.22	**0.003**	−49.90 ± 13.48	**0.005**
Ct. TMD [mg/cm^3^]	−25.27 ± 4.20	**<0.001**	−19.58 ± 6.04	**0.010**	−25.40 ± 6.79	**0.005**
Ct.Th [µm]	−21.17 ± 14.19	0.164	6.08 ± 7.67	0.449	−15.36 ± 19.20	0.444
Ct.Ar [mm^2^]	−4.17 ± 1.28	**0.007**	−0.14 ± 0.66	0.839	−2.21 ± 1.80	0.251
Ct.Po.V [standard] [mm^3^]	13.62 ± 4.49	**0.011**	15.18 ± 5.73	**0.027**	21.96 ± 5.84	**0.004**
Ct.Po.V [baseline-mapped] [mm^3^]	18.75 ± 2.45	**<0.001**	13.20 ± 4.45	**0.016**	22.36 ± 5.54	**0.003**
Ct.Po [standard] [%]	2.03 ± 0.56	**0.004**	1.55 ± 0.49	**0.012**	2.54 ± 0.65	**0.003**
Ct.Po [baseline-mapped] [%]	3.02 ± 0.49	**<0.001**	1.59 ± 0.44	**0.006**	3.10 ± 0.79	**0.003**
Ct.Po.Dm [standard] [µm]	19.50 ± 5.96	**0.007**	6.32 ± 4.14	0.161	2.62 ± 2.68	0.353
Ct.Po.Dm [baseline-mapped] [µm]	25.40 ± 5.80	**0.001**	8.41 ± 3.75	*0.052*	6.91 ± 3.09	*0.052*
Tb.N [mm^−1^]	−0.14 ± 0.05	**0.015**	−0.09 ± 0.04	**0.050**	0.04 ± 0.05	0.403
Tb.Th [µm]	6.83 ± 2.61	**0.024**	7.30 ± 3.03	**0.039**	−0.60 ± 2.26	0.796
Tb.Sp [µm]	37.58 ± 13.40	**0.017**	34.40 ± 13.60	**0.032**	−9.00 ± 14.15	0.541
Tb.Sp.SD [µm]	17.75 ± 5.60	**0.009**	14.20 ± 6.46	*0.056*	−12.80 ± 11.30	0.287
***Biomechanics***						
Stiffness, K [kN/mm]	−0.90 ± 1.99	0.659	−0.35 ± 7.98	0.668	−7.32 ± 2.80	**0.028**
App Modulus, E [MPa]	−4.26 ± 18.33	0.820	−1.61 ± 13.46	0.907	−79.53 ± 30.66	**0.029**
Estimated Failure Load, F [N]	−75.36 ± 85.27	0.396	−33.14 ± 31.33	0.318	−331.88 ± 120.73	**0.023**
Ct.LF_dist_ [%]	−3.19 ± 1.05	**0.011**	−1.78 ± 0.72	**0.036**	−1.95 ± 1.15	0.124

BL, baseline; FU, Follow-up; y, years; Δ, change; HR-pQCT, high resolution peripheral quantitative computed tomography; BMD, bone mineral density; Tt.BMD, total bone mineral density; Tb.BMD, trabecular BMD; Ct.BMD, cortical BMD; Ct.TMD, cortical tissue mineral density; Ct.Th, cortical thickness; Ct.Ar, cortical area; Ct.Po.V, intracortical pore volume; Ct.Po, intracortical porosity; Ct.Po.Dm, mean cortical pore diameter; Tb.N, trabecular number; Tb.Th, trabecular thickness, Tb.Sp, trabecular separation; Tb.Sp.SD, standard deviation of intertrabecular distances; Ct.LF dist, distal cortical load fraction; App Modulus, apparent modulus.

Cortical pore volume (Ct.Po.V), cortical porosity (Ct.Po), and cortical pore diameter (Ct.Dm) were reported as standard and baseline-mapped values. Intragroup differences between BL and FU HR-pQCT parameters were calculated via paired T-tests. Shown are means and standard errors (SEM). Significant p-values (p < 0.05) are marked in bold print, statistical trends are printed in italics.

[standard], the standard analysis method was used to compute the respective parameter: the cortical region was identified independently in both the baseline and follow-up images on scans that were matched on total cross-sectional area.

[baseline-mapped], baseline-mapped parameters were computed using a postprocessing method in which first a rigid transformation was applied that aligns the follow-up to the baseline scan and maps forward the baseline cortical border to the follow-up scan so that exactly the same region of the bone is measured as cortical bone, even if the bone may have undergone endocortical trabecularization during the FU time.

**Table 4:** Adjusted, annual percent changes (%) for HR-pQCT measured bone microarchitectural parameters at the ultradistal tibia and given for healthy, non-diabetic postmenopausal female controls (Co), T2D postmenopausal women without history of fragility fractures (DM), and T2D postmenopausal women with a positive history of fragility fractures (DMFx).

Ultradistal Tibia	Mean annual percent changes ^ł^ ± SEM			p- values		
	Co (n = 12)	DM (n = 10)	DMFX (n = 10)	Co vs. DM	Co *vs.* DMFx	DM *vs.* DMFx
***Basic HR-pQCT measures***						
Tt.BMD [%]	−0.68 ± 0.23	−0.43 ± 0.22	−0.92 ± 0.22	0.467	0.474	0.116
Tb.BMD [%]	0.18 ± 0.19	0.26 ± 0.19	0.15 ± 0.18	0.774	0.914	0.660
Ct.BMD [standard] [%]	−1.12 ± 0.29	−0.79 ± 0.28	−1.30 ± 0.28	0.458	0.685	0.206
Ct.BMD [baseline-mapped] [%]	−1.59 ± 0.29	−0.82 ± 0.28	−1.36 ± 0.27	*0.090*	0.600	0.173
Ct.TMD [%]	−0.57 ± 0.14	−0.42 ± 0.13	−0.57 ± 0.13	0.490	0.981	0.423
Ct.Th [%]	−0.50 ± 0.33	−0.01 ± 0.32	−0.04 ± 0.32	0.333	0.350	0.949
Ct.Ar [%]	−0.73 ± 0.29	−0.18 ± 0.28	−0.23 ± 0.28	0.224	0.259	0.893
Ct.Po.V [standard] [%]	3.79 ± 1.97	4.97 ± 1.93	6.61 ± 1.89	0.697	0.344	0.544
Ct.Po.V [baseline-mapped] [%]	6.68 ± 1.79	5.29 ± 1.74	7.32 ± 1.71	0.613	0.812	0.409
Ct.Po [standard] [%]	4.64 ± 2.10	5.25 ± 2.05	7.25 ± 2.01	0.848	0.408	0.486
Ct.Po [baseline-mapped] [%]	7.48 ± 1.90	5.88 ± 1.85	7.72 ± 1.81	0.584	0.932	0.478
Ct.Po.Dm [standard] [%]	2.12 ± 0.54	0.61 ± 0.52	0.09 ± 0.51	*0.076*	**0.018**	0.482
Ct.Po.Dm [baseline-mapped] [%]	2.82 ± 0.53	0.84 ± 0.52	0.50 ± 0.51	**0.021**	**0.007**	0.643
Tb.N [%]	−2.20 ± 0.62	−0.91 ± 0.61	0.96 ± 0.60	0.185	**0.002**	**0.037**
Tb.Th [%]	2.79 ± 0.82	1.38 ± 0.80	−0.69 ± 0.78	0.268	**0.008**	*0.072*
Tb.Sp [%]	2.45 ± 0.68	1.07 ± 0.67	−0.82 ± 0.65	0.197	**0.004**	*0.052*
Tb.Sp.SD [%]	2.38 ± 0.74	1.03 ± 0.73	−0.84 ± 0.71	0.244	**0.008**	*0.076*
***Biomechanics***						
Stiffness, K [%]	0.06 ± 0.33	−0.20 ± 0.32	−1.33 ± 0.32	0.614	**0.010**	**0.019**
App Modulus, E [%]	0.18 ± 0.32	−0.17 ± 0.31	−1.34 ± 0.31	0.483	**0.004**	**0.012**
Estimated Failure Load, F [%]	−0.08 ± 0.28	−0.24 ± 0.27	−1.13 ± 0.26	0.706	**0.017**	**0.025**
Ct.LF_dist_ [%]	−2.16 ± 0.42	−0.78 ± 0.41	−0.31 ± 0.40	**0.039**	**0.006**	0.419

HR-pQCT, high resolution peripheral quantitative computed tomography; BMD, bone mineral density; Tt.BMD, total bone mineral density; Tb.BMD. trabecular BMD; Ct.BMD, cortical BMD; Ct.TMD, cortical tissue mineral density; Ct.Th, cortical thickness; Ct.Ar, cortical area; Ct.Po.V, intracortical pore volume; Ct.Po, intracortical porosity; Ct.Po.Dm, mean cortical pore diameter; Tb.N, trabecular number; Tb.Th, trabecular thickness, Tb.Sp, trabecular separation; Tb.Sp.SD, standard deviation of intertrabecular distances; Ct.LF dist, distal cortical load fraction; App Modulus, apparent modulus.

Shown are adjusted means with standard errors (SEM). Significant p-values (p < 0.05) are marked in bold print, statistical trends are printed in italics. Ł adjusted for race and Δ BMI.

[standard], the standard analysis method was used to compute the respective parameter: the cortical region was identified independently in both the baseline and follow-up images on scans that were matched on total cross-sectional area.

[baseline-mapped] = baseline-mapped parameters were computed using a postprocessing method in which first a rigid transformation was applied that aligns the follow-up to the baseline scan and maps forward the baseline cortical border to the follow-up scan so that exactly the same region of the bone is measured as cortical bone, even if the bone may have undergone endocortical trabecularization during the FU time.

DM and Co subjects similarly exhibited significant losses in cortical BMD (Controls: −5.7% Ct.BMD, p < 0.001, DM subjects: −3.9% Ct.BMD, p = 0.003), and cortical bone microstructure including cortical TMD (Controls: +19.1% Ct.PoV, p = 0.011; +24.7% Ct.Po, p = 0.004; −2.7% Ct.TMD, p < 0.001; DM subjects: +23.2% Ct.PoV, p = 0.027; +22.3% Ct.Po, p = 0.012; −2.1% Ct.TMD, p = 0.010). Additionally, the Co group exhibited significant decreases in total BMD (−3.8%, Tt.BMD, p = 0.001) and Ct.Ar (−3.9% Ct.Ar, p = 0.007), and a significant increase in cortical pore diameter (Co: +8.8% Ct.Po.Dm, p = 0.007).

In contrast to the DMFx group, both DM and Co subjects exhibited significant changes in most trabecular microarchitectural parameters: in both Co and DM groups there was a significant decrease in trabecular number accompanied by a significant increase in trabecular thickness and trabecular spacing (Co: +8.8% Tb.Th, p = 0.024, + 7.5% Tb.Sp, p = 0.017; DM: +8.4% Tb.Th, p = 0.039, 6.0% Tb.Sp, p = 0.032).

Annualized percent changes were calculated and compared between groups. At the ultradistal tibia, annualized percent changes in density parameters (Tt.BMD, Tb.BMD, Ct.BMD) and in cortical bone parameters including Ct.Po, Ct.Po.V, and Ct.TMD were similar between all three groups (p > 0.05, **Table 4**). Despite comparable Ct.Po.Dm at baseline, the rate of change in cortical pore diameter was significantly different between groups: the cortical pore diameter in the Co group increased about 23.5× more annually compared to DMFx subjects (p = 0.018), and 3.5× more annually compared to DM subjects (p = 0.076). With respect to the trabecular bone compartment controls showed significantly larger annualized changes in trabecular thickness, spacing, and trabecular heterogeneity compared to DMFx subjects (0.008 ≤ p ≤ 0.002). A sensitivity analysis using a linear regression model with an additional adjustment for age yielded consistent results compared to the original analysis (with only race and change in BMI adjustments, **Supplemental Table 6**). Only for total and trabecular BMD new significant and trending significant differences were observed, indicating that DMFx subjects annually lost significantly more total BMD (p = 0.028) and trending more trabecular BMD (p = 0.097) than DM subjects.

At the ultradistal radius we observed over the entire follow-up period a similar pattern of longitudinal BMD loss and loss in cortical microstructure as noted for the ultradistal tibial scan site (**Supplemental Table 2**). However, intragroup changes at the ultradistal radius were in general less pronounced than at the tibial site and did not always reach statistical significance. Significant absolute changes in trabecular bone structure within each group were also not present. Similar to the findings seen at the ultradistal tibia scan site, annualized percent changes of BMD (Tt.BMD, Ct.BMD, Tb.BMD, Ct.TMD) and of cortical microstructure at the ultradistal radius were also comparable among the three groups (**Supplemental Table 3**), besides a significantly larger annual decrease in Tt.BMD in DMFx relative to DM subjects (p = 0.027). The annual rate of cortical pore diameter change was not significantly different among the three groups as were annualized changes in trabecular structure and geometry.

### Longitudinal Changes in Bone Strength

At the ultradistal tibial scan site, bone strength of DMFx subjects decreased significantly over the entire follow-up period in almost all bone strength indices (DMFx: −5.9% stiffness K, p = 0.028, −4.9% failure load F, p = 0.023, −5.9% apparent modulus, p = 0.029), except for cortical load fraction where declines did not reach statistical significance (−4.4 Ct.LF, p = 0.124). In controls and DM subjects, no significant declines in the bone strength indices stiffness, apparent modulus, and estimated failure load were observed during their respective entire FU-times, but a significant decrease in cortical load fraction was noted (Co: −7.5.% Ct.LF, p = 0.011; DM: −3.9%, p = 0.036).

When comparing annualized percent changes between groups, bone strength indices decreased annually 7.4 to 22.2× more in DMFx subjects relative to controls (DMFx *vs.* Co: −22.2× stiffness K, p = 0.010; −14.1× failure load F, p = 0.017, −7.4× apparent modulus E, p = 0.004; −7.0× cortical load fraction Ct.LF, p = 0.006) (45), and 4.7 to 7.8 times more in DMFx *versus* DM subjects (DMFx *vs.* DM: −6.7× stiffness K, p = 0.019, −4.7× failure load F, p = 0.025, −7.8× apparent modulus E, p = 0.012).

Similar to the tibia results, DMFx subjects exhibited at the ultradistal radius during their entire FU period significant declines in almost all bone strength indices (DMFx: −8.3% stiffness, p = 0.043, −4.3% failure load, p = 0.042, −11.3% apparent modulus, p = 0.034), except for cortical load fraction where declines did again not reach statistical significance (−0.1% Ct.LF, p = 0.968). Unlike at the tibia, DM subjects did display at the radial scan site significant declines in stiffness (−3.4%, p = 0.048), failure load (−3.0%, p = 0.053), and apparent modulus (−5.1%, p = 0.005) over the entire FU-period. Their +3.1% increase in cortical load fraction did not reach statistical significance (p = 0.176). In controls, smaller longitudinal decreases in stiffness (−4.4%), in apparent modulus (−5.5%) and failure load (−4.0%) were noted without reaching statistical significance. Annualized rates of decline in biomechanical indices (K, F, E) at the ultradistal radius were about 1.8 to 1.9× higher in DMFx *versus* Co subjects and about 2.6 to 4.8× higher in DMFx *versus* DM subjects, however none of these comparisons were statistically significant.

### Comparison of Standard and Baseline-Mapped Cortical Measurement Methods

Both methods utilized to quantify cortical porosity and cortical pore volume demonstrated for each of the three groups significant increases over time in Ct.Po and Ct.Po.V at both the ultradistal tibia (0.001 ≤ p ≤ 0.027) and ultradistal radius (0.001 ≤ p ≤ 0.066) (**Table 3** and **Supplementary Table 2**). With both methods, Ct.Po and Ct.Po.V increased at the ultradistal tibia at similar annual rates in all three groups: using the standard method, Ct.Po [standard] increased annually by 7.3, 5.3, and 4.6% in DMFx, DM, and Co subjects, respectively, while using the baseline-mapped method, Ct.Po [baseline-mapped] increased annually by 7.7%, 5.9% and 7.5% in DMFx, DM and Co subjects, respectively. We also tested whether the annual percent changes in Ct.Po and in Ct.Po.V as assessed by the two different methods were significantly different within each group. We found that only in the Co group annual percent changes in Ct.Po and Ct.Po.V were significantly larger with baseline-mapping compared to the standard technique (+4.6% Ct.Po [standard] *versus* +7.5% Ct.Po [baseline-mapped], p = 0.002; +3.8% Ct.Po.V [standard] *versus* +6.7% Ct.Po.V [baseline-mapped] p = 0.007), as were annual changes in Ct.Po.Dm (+2.1% Ct.Po.Dm [standard] *versus* +2.8% Ct.Po.Dm [baseline-mapped] p = 0.043) and in Ct.BMD (−1.1% Ct.BMD [standard] *versus* −1.6% Ct.BMD [baseline-mapped] p = 0.001). In contrast, in the DM and DMFx groups, annualized percent Ct.Po and Ct.Po.V, Ct.BMD, and Ct.Po.DM changes were comparable using either method (p > 0.05).

Results at the ultradistal radius using both methods mirrored those at the ultradistal tibia. With both methods, Ct.Po and Ct.Po.V increased at the ultradistal radius at similar annual rates in all three groups. When we assessed whether the annual percent changes in Ct.Po and in Ct.Po.V as quantified by the two different methods were significantly different within each group, we found that as at the tibia, only in the Co group annual percent changes in Ct.Po, in Ct.Po.V, and in Ct.BMD were significantly larger with baseline-mapping compared to the standard technique (Ct.Po.V [standard] +12.0% *vs.* Ct.Po.V [baseline-mapped] +15.5%, p = 0.026; Ct.Po [standard] +12.8% *vs.* Ct.Po [baseline-mapped] +17.8%, p = 0.004). In the DM and DMFx groups instead, annualized percent Ct.Po, Ct.Po.V Ct.Po.V, Ct.BMD, and Ct.Po.DM changes were at the ultradistal radius comparable with either method (p > 0.05).

## Discussion

Epidemiological studies have demonstrated an increased risk for fragility fractures in individuals with T2D despite normal or even elevated bone mineral density measurements by DXA (4, 5, 7), suggesting that diabetes-induced alterations in bone material properties and/or microarchitecture increase fracture risk independent of BMD (6). Accordingly, recent cross-sectional studies provided first evidence that cortical deficits such as a higher cortical porosity may be one of the leading microstructural pathomorphologies of diabetic bone disease (10, 16, 17). However, the temporal evolution of cortical porosity, bone microarchitecture, and micro-scale bone biomechanics in individuals with T2D are still unknown (46). Therefore, we designed this longitudinal study in order to investigate the temporal evolution of cortical porosity and of associated pore parameters *via* HR-pQCT and to study the longitudinal alterations of bone microarchitecture and of bone strength during the course of T2D bone disease.

One of the main findings of this exploratory study was that T2D postmenopausal women with (DMFx) or without a history of a fragility fracture (DM) exhibited significant increases in cortical porosity and cortical pore volume over the entire 4–5-year follow-up period. However, the annual percent increases in cortical porosity and in cortical pore volume observed in DMFx and DM subjects were in the range of non-diabetic postmenopausal controls (Co) at the ultradistal tibia. At first glance these results seem surprising. Specifically, as DMFx subjects exhibited at baseline significantly higher porosity and pore volumes than DM subjects and numerically higher porosity and pore volume than non-diabetic Co subjects one would have hypothesized that DMFx women, who can be considered a high-risk fracture group (20), would exhibit a greater annual increase in cortical porosity than DM subjects and non-diabetic controls. Instead we found comparable annual rates of cortical porosity and cortical pore volume increases among the three groups. These rates and magnitudes remained comparable between groups no matter if cortical contours were independently determined on baseline and follow-up images or if baseline contours were forward-mapped to the follow-up image. The correspondence between standard and baseline-mapped results is important because it confirms that the porosity parameter changes detected in our study are not skewed or erroneous due to the exclusion of “trabecularized” cortical bone on the follow-up image. The fact that we detected comparable annual rates of porosity and pore volume between groups may be due to the relatively small sample size of our groups. However, the observed annual cortical porosity changes in our small study ranged within in the same magnitude that is reported for healthy postmenopausal women from 5-year longitudinal data derived from the large population-based CaMos study for the respective age group (6^th^ decade) (47). Another potential explanation for the comparable rate of cortical porosity increase despite divergent baseline porosity may be that cortical porosity development in T2D diabetic bone disease may not necessarily follow a linear pattern, and that periods of escalated pore growth may be followed by times of slower pore growth, a phase which we might have randomly hit in the time window that we imaged in this longitudinal study. As this is to our knowledge the first *in vivo* human study examining the temporal course of cortical porosity and cortical pore size development in T2D diabetic bone disease *via* HR-pQCT, other clinical studies in T2D diabetic bone disease were not available for comparison. However, population-based data on healthy, non-diabetic postmenopausal women corroborate that cortical porosity formation seems not to occur linearly but rather multimodally throughout lifetime and that phases of more rapid cortical porosity formation are preceded or followed by times of slower porosity increase (47). Moreover, from other metabolic bone diseases such as chronic kidney disease—a mineral and bone disorder (CKD-MBD) which shares certain commonalities with diabetic bone disease (48)—we know that cortical porosity development in CKD-MBD does not behave linearly, and that phases of slow and accelerated cortical pore formation can occur during the course of disease (49). What exactly triggers the slowing or acceleration of cortical pore formation and growth remains unsolved, but may partly result from the complex interplay between trabecular and cortical bone (49) or from disease severity or management (50). In our study, DMFx patients had on average a 5-year longer duration of diabetes and exhibited partly secondary diabetic complications, specifically microvascular disease such as neuropathy (4/10) and diabetic retinopathy (2/10). This points to a slightly higher baseline disease severity/microvascular complication load in DMFx subjects relative to DM subjects, who did not show any signs of secondary diabetic complications. Given that recent cross-sectional findings by Samelson et al. and Shanbogue et al. linked longer duration of T2D and the presence of microvascular secondary diabetic complications with higher baseline cortical porosity (17) (51), it seems likely that our DMFx subjects had already experienced before enrolment a severity-triggered bout of cortical porosity formation resulting in a higher baseline porosity. This higher baseline porosity in DMFx subjects was then followed during our follow-up study window by a course of smaller pore growth—comparable to Ct.Po increases observed in Co and DM groups. However, further large-scale and long-term clinical studies and trials involving repeated HR-pQCT measurements in T2D patients are needed to validate our findings and to evaluate the triggers and treatments leading to slowing or acceleration of cortical pore formation and growth in T2D diabetic bone disease.

**Figure 1:**
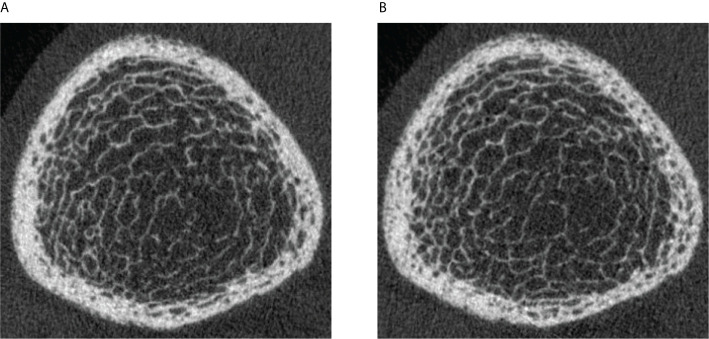
Legend: **(A)** HR-pQCT scan (ultradistal tibia) of a 63-year-old postmenopausal woman with a 11-year history of type 2 diabetes mellitus and a history of right metatarsal fragility fracture prior to enrollment. Significant cortical porosity and trabecular heterogeneity are demonstrated. **(B)** HR-pQCT scan (ultradistal tibia) of the same patient, 3.6 years later during the follow-up visit: note the increase in cortical porosity, predominantly in the midcortical and periosteal parts of the cortex.

In order to better understand the spatial pattern of longitudinal cortical pore expansion in T2D bone disease, we employed two different longitudinal cortical mapping techniques—the standard and the baseline-mapping method. This is advantageous as both methods can provide useful additional information on spatial bone changes occurring locally at the cortico-trabecular interface, an important transition zone and area of active bone remodeling (52). Particularly in T2D bone disease, where the structural and spatial mechanisms of cortical bone loss are still unclear and where increased intracortical and endocortical porosity exist (11) with its potential higher propensity to cause endocortical trabecularization, application of both techniques can help clarify true endocortical porosity formation and avoid skewed or erroneous results due to the exclusion of “trabecularized” cortical bone on the follow-up image. In our study, we used both techniques and found with both methods significant increases in cortical porosity in all three groups during the entire follow-up time. When examining the magnitude of cortical porosity change captured by each method within each group, we found that for non-diabetic controls annual percent changes in cortical porosity, pore volume, and cortical BMD were significantly greater using the baseline-mapping technique than when the standard mapping technique was applied. These results concur with previous literature indicating that the endocortical zone is a prominent area for cortical bone loss in non-diabetic postmenopausal women (53). In the DM and DMFx groups, we observed instead that changes in mean annual cortical porosity, cortical pore volume, diameter, and cortical BMD were similar with either technique. These findings extend our prior observations (11) that the predominant bone loss and cortical porosity changes in T2D bone disease may not primarily occur at the endosteal zone but may rather occur in the midcortical and periosteal zones of the cortex. Visual inspection of our baseline and follow-up images confirmed that newly formed or enlarged cortical pores were mainly in the midcortical and periosteal layers in T2D individuals (**Figure 1**). This suggests that mechanisms of pore growth in T2D may impact primarily midcortical and periosteal rather than endocortical zones of the cortex.

Another important observation of our study was that the annual decline in bone strength was significantly and much larger in the DMFx subjects relative to Co and DM subjects. Interestingly, this significant annual decline in bone strength in DMFx subjects occurred although annual rates of cortical porosity and volume changes were comparable relative to Co and DM subjects. Our findings are in line with the clinical notion that DMFx women had—unlike DM subjects—all sustained diabetic fragility fractures in the past (prior to enrollment), and therefore can be regarded as a clinical high-risk fragility group. From a biomechanical perspective, this increased decline in bone strength in the DMFx group may be explained in part by the following mechanisms: DMFx patients did display at baseline and at follow-up the highest cortical porosity and pore volumes. Several *in vitro* studies have shown that cortical bone strength depends not only on its porosity (54, 55), but also on pore size (56), pore variation (57), and spatial distribution of porosity within the cortex (58). Thus, increasing pore size has been linked to a deterioration in cortical biomechanical competence (56) most likely by lowering the available matrix for microcrack propagation (59) and by providing larger stress concentrations for microcrack initiation (60). The observed reduction in bone strength in DMFx may also be related to the fact that DMFx patients displayed a pre-aged bone phenotype. In fact, the magnitude of cortical porosity found at baseline in DMFx subjects exceeded the amount of cortical porosity normally not even reached by postmenopausal controls in their 9^th^ decade of life (47), suggesting an accelerated-aged bone phenotype in the DMFx group. As aging is considered the most significant risk factor for fractures (61), and goes along with up to 10% degradation in biomechanical competence per decade (62) the accelerated-aged bone phenotype observed in our DMFx group may by another relevant factor contributing to the observed increased decline in bone strength in DMFx subjects.

Another interesting finding of our study was that serum CTX levels were significantly lower in both diabetic groups compared to controls. Although not significant, P1NP baseline serum levels were numerically lower in both diabetic groups compared to controls. These findings suggest a reduced bone turnover in both diabetic groups and are in line with numerous studies in the literature indicating a state of low bone turnover associated with T2D (63, 64). Although we did not have any bone turnover markers available at the follow-up visit, we observed particularly in the DMFx group larger declines in bone strength relative to controls during the follow-up time. Given the initially low bone turnover profile in this patient group, our findings support the current mechanistic hypothesis that low bone turnover in diabetes may cause insufficient bone renewal with unrepaired micro-cracks leading to an increased bone fragility (63).

With respect to trabecular bone, DMFx patients exhibited at baseline—although not significant—on average a 12% lower tibial trabecular BMD and numerically lower trabecular numbers than controls. Compared to normative cohort data, DMFx patients displayed thus at the tibia in their 6^th^ decade mean baseline trabecular parameters normally found in postmenopausal women in their later 70s (47). This suggests that DMFx patients displayed relative to the control group at baseline not only an accelerated-aged cortical bone phenotype—as indicated above by the high amount of baseline cortical porosity—but also an accelerated-aged trabecular bone profile. When looking at the trabecular changes over time in both groups, we noted significant differences in trabecular changes between DMFx and Co subjects. While controls exhibited trabecular bone changes more indicative of age-related endocortical remodeling and trabecularization (loss in trabeculae with corresponding significant increases in trabecular thickness, spacing, and heterogeneity), DMFx patients in turn showed significant increases in trabeculae with concomitant decreases in trabecular thickness, spacing, and heterogeneity. These latter trabecular changes together with the cortical changes noted in the DMFx group are suggestive of a relative preservation of the trabecular bone structure in DMFx subjects over the follow-up period with the main bone loss rather occurring cortically in this accelerated-aged patient group. These findings are in line with the notion, that bone loss becomes predominantly cortical with advancing age (56), but have to be cautiously interpreted, given the limited spatial resolution of the first generation HR-pQCT scanner used in this study and the resulting increased susceptibility of trabecular parameters to partial volume errors (65).

Our study has several strengths. First, our study is the first longitudinal clinical study investigating *via in vivo* high-resolution imaging the longitudinal evolution of bone strength and cortical bone microarchitecture in T2D individuals. In addition, this study evaluates in detail the impact of bone changes occurring at the cortico-trabecular interface (36). Another advantage of our study is that the baseline-mapped technique applied three-dimensional image registration which allowed for analysis of the common bone region free of angular deviations (66) and avoided also potential errors related to periosteal changes that might occur over longer observation intervals. Additionally, we reported our results normalized annually although our data were acquired from 4- to 5-year follow-ups. This ensures a better comparability with other data and with future longitudinal studies. Moreover, we included an additional T2D group with a positive history of fragility fracture (DMFx group) prior to enrolment. This group can be considered—as noted before (20)—as a high-risk T2D group, which is specifically susceptible to fragility fractures and which may clinically likely benefit most from fracture-preventive pharmacologic treatment and therefore warrants thorough scientific characterization.

Our study has several limitations. First, this longitudinal study was mainly exploratory in nature and the number of study participants was relatively small (n = 10–12 subjects per group). Secondly, HR-pQCT only allows assessment of peripheral skeletal sites such as tibia and radius but cannot be used to assess bone microarchitecture at the central skeleton such as at the proximal femur or spine. Therefore, it is not possible to extrapolate our findings to the central skeleton of diabetic patients. More studies are needed to investigate how central bone microarchitecture changes over time in diabetic bone disease. A third limitation of our study was that we did not perform a blood draw and laboratory serum analyses at follow-up. Therefore, markers of bone metabolism were only available at baseline. However, we were able to extract general important clinical bloodwork information for the follow-up visit from participants’ routine primary care laboratory reports dating from within the last 2 months prior to the follow-up study date. A fourth shortcoming of our study is that despite providing a nominal isotropic resolution of 80 µm and despite its validation against micro-CT for advanced cortical parameters (67), cortical porosity as assessed *via* HR-pQCT measurements can only be quantified directly to a pore diameter of 100 µm. However, cortical tissue mineral density is an inverse surrogate marker of subresolution microporosity (44) and was therefore included and reported in our study. Further studies using the increased resolution of second-generation HR-pQCT scanners and additional *ex vivo* micro-CT studies in T2D bone samples are necessary to shed more light on the role of microporosity in diabetic bone fragility.

In summary, our observational longitudinal data suggest that cortical bone loss and increases in cortical porosity in T2D bone disease may occur mainly in the midcortical and periosteal zones of the cortex, while they seem predominantly localized along the endocortical surface in postmenopausal controls. Furthermore, our findings suggest that, despite different baseline levels in cortical porosity, T2D women with and without fragility fractures experienced pore growth at an annual rate similar to non-diabetic controls. However, the annual loss in biomechanical indices such as cortical stiffness, failure load, and apparent modulus was by far greatest in T2D women with a history of a fragility fractures who suffered from T2D on average for 11 years prior to enrollment and exhibited signs of secondary diabetic complications. This suggests a potentially non-linear course of cortical porosity development in T2D: major porosity development may occur rather early in the course of disease (or potentially as a function of disease severity), followed by a smaller steady annual increase in porosity which in turn can still have a detrimental effect on bone strength—depending on the amount of early cortical pre-damage. Further large-scale prospective studies are needed to elucidate the important covariates and triggers for slowing or acceleration of cortical pore formation and growth in T2D diabetic bone disease.

## Data Availability Statement

The datasets presented in this article are not readily available because of ethical and privacy restrictions. Requests to access the datasets should be directed to UH *via* ursula.heilmeier@ucsf.edu.

## Ethics Statement

This study involved human participants and was reviewed and approved by the UCSF IRB committee. All participants provided their written informed consent to participate in this study.

## Author Contributions

UH contributed to the manuscript in the following ways: study conception and design, study set up and conduct, data collection, analysis and interpretation of data, statistical modeling, drafting of manuscript. The following authors contributed as described: CP (data analysis, revising manuscript content), GBJ (statistical analysis, revising manuscript content), ND/JY/ST/KD (data acquisition, revising manuscript content), JCG (data collection and analysis, revising manuscript content), AJB and TML (study conduct and data analysis baseline visit, revising manuscript content), GJK (study design, data analysis, revising manuscript content). UH and GJK take responsibility for the integrity of the data analysis. All authors contributed to the article and approved the submitted version.

## Funding

This study was supported by National Institutes of Health grants NIH R01 AG17762, R03 AR064004, and K01 AR056734 to GJK, RC1 AR058405 to TML, and R01 AR060700 to AJB, and R01 AR068456 to JCG and AJB, by UCSF Department of Radiology Seedgrant No. #27-13 (UH), and by the University of California Berkeley, Undergraduate Research Apprentice Program (URAP Berkeley) to CP.

## Conflict of Interest

AJB has served as a consultant for Ultragenyx Pharmaceutical Inc. and Mereo BioPharma Group PLC, and received research support from Ultragenyx. TML, GJK, JCG, and AJB received grant money from NIH-NIAMS. UH received travel funds from ASBMR.

The remaining authors declare that the research was conducted in the absence of any commercial or financial relationships that could be construed as a potential conflict of interest.
