# Supplementary material for: Longitudinal Evolution of Bone Microarchitecture and Bone Strength in Type 2 Diabetic Postmenopausal Women With and Without History of Fragility Fractures—A 5-Year Follow-Up Study Using High Resolution Peripheral Quantitative Computed Tomography
Source: Front Endocrinol (Lausanne). 2021 Mar 16;12:599316. doi: 10.3389/fendo.2021.599316 (PMC8008748; doi:10.3389/fendo.2021.599316)
Supplement: Supplementary file 1 [file DataSheet_1.docx]

Supplementary Material

# Supplementary Tables

| **Supplemental Table 1. Baseline bone microarchitectural parameters measured via HR-pQCT at the ultradistal radius** given for healthy, non-diabetic, postmenopausal female controls (Co), T2D postmenopausal women without history of fragility fractures (DM), and T2D postmenopausal women with a positive history of fragility fractures (DMFx). Intergroup differences were assessed using linear regression models adjusted for race. Shown are race-adjusted means and standard errors (SEM). Boldface indicates significant difference (p<0.05). | | | |
| --- | --- | --- | --- |
|  | **Baseline (adjusted) means ± SEM** | | |
| **Ultradistal Radius** | **Co (n=11)** | **DM (n=9)** | **DMFX (n=7)** |
| ***Basic HR-pQCT measures*** |  |  |  |
| Tt.BMD [mg/cm^3^] | 290.4 ± 21.35 | 295.4 ± 23.34 | 289.1 ± 23.29 |
| Tb.BMD [mg/cm^3^] | 155.7 ± 11.81 | 140.5 ± 12.92 | 152.6 ± 12.88 |
| Ct.BMD [mg/cm^3^] | 938.6 ± 18.13 | 921.9 ± 19.39 | **876.9 ± 21.00 ^$^** |
| Ct. TMD [mg/cm^3^] | 972.4 ± 14.18 | 968.8 ± 15.16 | 942.6 ± 16.43 |
| Ct.Th [mm] | 0.78 ± 0.06 | 0.86 ± 0.06 | 0.81 ± 0.06 |
| Ct.Ar [mm^2^] | 50.3 ± 2.87 | 54.6 ± 3.07 | 53.2 ± 3.32 |
| Ct.Po.V [mm^3^] | 9.43 ± 2.37 | 13.41 ± 2.54 | **17.97 ± 2.75 ^$^** |
| Ct.Po [%] | 2.34 ± 0.64 | 3.24 ± 0.68 | **4.77 ± 0.74 ^$^** |
| Ct.Po.Dm [µm] | 184.0 ± 7.53 | 178.6 ± 8.06 | 194.0 ± 8.73 |
| Tb.N [mm^-1^] | 1.82 ± 0.09 | 1.85 ± 0.10 | 1.84 ± 0.11 |
| Tb.Th [µm] | 70.8 ± 3.22 | 62.3 ± 3.44 | 68.0 ± 3.73 |
| Tb.Sp [µm] | 490.6 ± 29.33 | 489.5 ± 31.36 | 485.0 ± 33.97 |
| Tb.Sp.SD [µm] | 209.0 ± 21.49 | 208.9 ± 22.98 | 227.2 ± 24.89 |
| ***Biomechanics*** |  |  |  |
| Stiffness, K [kN/mm] | 48.0 ± 3.04 | 47.3 ± 3.26 | 47.9 ± 3.52 |
| App Modulus, E [MPa] | 1186.4 ± 92.97 | 1186.9 ± 99.40 | 1074.5 ± 107.68 |
| Estimated Failure Load, F [N] | 2605.9 ± 150.21 | 2581.3 ± 160.61 | 2531.1 ± 173.98 |
| Ct.LF _dist_ [%] | 46.8 ± 2.67 | 52.2 ± 2.86 | 45.0 ± 3.09 |
| HR-pQCT = high resolution peripheral quantitative computed tomography; T2D = type 2 diabetes; BMD = bone mineral density; Tt.BMD = total bone mineral density; Tb.BMD = trabecular BMD; Ct.BMD = cortical BMD; Ct.TMD = cortical tissue mineral density; Ct.Th = cortical thickness; Ct.Ar = cortical area; Ct.Po.V = intracortical pore volume; Ct.Po = intracortical porosity; Ct.Po.Dm = mean cortical pore diameter; Tb.N = trabecular number; Tb.Th = trabecular thickness, Tb.Sp = trabecular separation; Tb.Sp.SD = standard deviation of intertrabecular distances; Ct.LF dist = distal cortical load fraction; App Modulus = apparent modulus;  * p< 0.05 DM vs. DMFx.  **^$^**  signifies p< 0.05 vs. Co group. | | | |

| **Supplemental Table 2. Longitudinal absolute changes in HR-pQCT-derived bone microarchitectural parameters and biomechanical parameters measured at the ultradistal radius.** Data are shown per group and over entire follow-up time period of each group, respectively for healthy, non-diabetic, postmenopausal female controls (Co), T2D postmenopausal women without history of fragility fracture (DM) and T2D postmenopausal women with a positive history of fragility fractures (DMFx). Cortical pore volume (Ct.Po.V), cortical porosity (Ct.Po) and cortical pore diameter (Ct.Po.Dm) were reported as standard and baseline-mapped values. Intragroup differences between BL and FU HR-pQCT parameters were calculated via paired t-tests. Shown are means and standard errors (SEM). Significant p-values (p<0.05) are marked in bold print, statistical trends are printed in italics. | | | | | | |
| --- | --- | --- | --- | --- | --- | --- |
|  | **Absolute Δ over**  **5.2 ± 0.3 y FU-time** | | **Absolute Δ over**  **4.9 ± 0.5 y FU-time** | | **Absolute Δ over**  **4.0 ± 0.8 y FU-time** | |
| **Ultradistal Radius** | **Co (n=11)** | | **DM (n=9)** | | **DMFx (n=7)** | |
|  | **Means** **± SEM** | **p** | **Means** **± SEM** | **p** | **Means** **± SEM** | **p** |
| ***Basic HR-pQCT measures*** |  |  |  |  |  |  |
| Tt.BMD [mg/cm^3^] | -9.35 ± 3.21 | **0.016** | -2.17 ± 3.03 | 0.495 | -12.91 ± 3.23 | **0.007** |
| Tb.BMD [mg/cm^3^] | -1.73 ± 1.80 | 0.360 | 2.04 ± 1.81 | 0.300 | 0.58 ± 0.51 | 0.299 |
| Ct.BMD [mg/cm^3^] | -38.15 ± 7.18 | **<0.001** | -24.53 ± 12.72 | *0.090* | -27.71 ± 3.92 | **<0.001** |
| Ct. TMD [mg/cm^3^] | -21.88 ± 5.33 | **0.002** | -13.03 ± 8.58 | 0.167 | -16.24 ± 2.08 | **<0.001** |
| Ct.Th [µm] | -12.02 ± 6.47 | *0.093* | -18.96 ± 13.16 | 0.188 | -31.97 ± 16.98 | 0.109 |
| Ct.Ar [mm^2^] | -0.68 ± 0.38 | 0.106 | 0.41 ± 0.75 | 0.602 | -1.45 ± 0.93 | 0.172 |
| Ct.Po.V [standard] [mm^3^] | 4.23 ± 0.75 | **<0.001** | 4.42 ± 1.95 | *0.054* | 2.92 ± 1.35 | *0.074* |
| Ct.Po.V [baseline-mapped] [mm^3^] | 4.60 ± 0.94 | **0.001** | 4.45 ± 1.90 | **0.048** | 3.35 ± 0.85 | **0.011** |
| Ct.Po [standard] [%] | 1.20 ± 0.24 | **0.001** | 1.01 ± 0.48 | *0.066* | 0.89 ± 0.29 | **0.022** |
| Ct.Po [baseline-mapped] [%] | 1.67 ± 0.35 | **0.001** | 1.48 ± 0.57 | **0.032** | 1.17 ± 0.34 | **0.018** |
| Ct.Po.Dm [standard] [µm] | 6.66 ± 3.92 | 0.120 | 2.94 ± 5.91 | 0.632 | 4.13 ± 7.85 | 0.618 |
| Ct.Po.Dm [baseline-mapped] [µm] | - 1. ± 4.11 | 0.135 | -2.17 ± 6.67 | 0.753 | 6.58 ± 7.43 | 0.416 |
| Tb.N [mm^-1^] | -0.07 ± 0.05 | 0.156 | 0.01 ± 0.04 | 0.863 | 0.04 ± 0.05 | 0.430 |
| Tb.Th [µm] | 2.72 ± 2.00 | 0.202 | 1.11 ± 0.95 | 0.276 | -1.43 ± 1.76 | 0.447 |
| Tb.Sp [µm] | 30.54 ± 15.85 | *0.083* | -0.11 ± 11.07 | 0.992 | -9.57 ± 13.61 | 0.508 |
| Tb.Sp.SD [µm] | 28.91 ± 14.54 | *0.075* | 1.33 ± 5.62 | 0.818 | -1.29 ± 5.61 | 0.826 |
| ***Biomechanics*** |  |  |  |  |  |  |
| Stiffness, K [kN/mm] | -2.07 ± 1.30 | 0.142 | -1.58 ± 0.68 | **0.048** | -3.59 ± 1.41 | **0.043** |
| App Modulus, E [MPa] | -59.78 ± 28.44 | *0.062* | -63.41 ± 16.26 | **0.005** | -104.12 ± 38.06 | **0.034** |
| Estimated Failure Load, F [N] | -105.18 ± 61.87 | 0.120 | -74.74 ± 32.98 | *0.053* | -158.26 ± 61.45 | **0.042** |
| Ct.LF _dist_ [%] | 0.90 ± 1.19 | 0.468 | 1.62 ± 1.91 | 0.176 | -0.06 ± 1.49 | 0.968 |
| HR-pQCT = high resolution peripheral quantitative computed tomography; T2D = type 2 diabetes; BL= baseline; FU = Follow-up; y= years; Δ= change; BMD = bone mineral density; Tt.BMD = total bone mineral density; Tb.BMD = trabecular BMD; Ct.BMD = cortical BMD; Ct.TMD = cortical tissue mineral density; Ct.Th = cortical thickness; Ct.Ar = cortical area; Ct.Po.V = intracortical pore volume; Ct.Po = intracortical porosity; Ct.Po.Dm = mean cortical pore diameter; Tb.N = trabecular number; Tb.Th = trabecular thickness, Tb.Sp = trabecular separation; Tb.Sp.SD = standard deviation of intertrabecular distances; Ct.LF dist = distal cortical load fraction; App Modulus = apparent modulus;  [standard] = the standard analysis method was used to compute the respective parameter: the cortical region was identified independently in both the baseline and follow-up images on scans that were matched on total cross-sectional area.  [baseline-mapped] = baseline-mapped parameters were computed using a postprocessing method in which first a rigid transformation was applied that aligns the follow-up to the baseline scan and maps forward the baseline cortical border to the follow-up scan so that exactly the same region of the bone is measured as cortical bone, even if the bone may have undergone endocortical trabecularization during the FU-time. | | | | | | |

| **Supplemental Table 3. Adjusted, annual percent changes (%) for HR-pQCT measured bone microarchitectural parameters at the ultradistal radius.** Results are given for non-diabetic, postmenopausal female controls (Co), T2D postmenopausal women without history of fragility fractures (DM), and T2D postmenopausal women with a positive history of fragility fractures (DMFx). Shown are adjusted means with standard errors (SEM). Significant p-values (p<0.05) are marked in bold print, statistical trends are printed in italics. | | | | | | |
| --- | --- | --- | --- | --- | --- | --- |
|  | **Mean annual percent changes ^ł^ ± SEM** | | | **p- values** | | |
| **Ultradistal Radius** | **Co (n=11)** | **DM (n=9)** | **DMFX (n=7)** | **Co vs.**  **DM** | **Co vs. DMFx** | **DM vs. DMFx** |
| ***Basic HR-pQCT measures*** |  |  |  |  |  |  |
| Tt.BMD [%] | -0.92 ± 0.28 | -0.01 ± 0.29 | -1.04 ± 0.30 | *0.055* | 0.777 | **0.027** |
| Tb.BMD [%] | -0.23 ± 0.28 | 0.25 ± 0.30 | 0.16 ± 0.31 | 0.305 | 0.382 | 0.845 |
| Ct.BMD [standard] [%] | -0.87 ± 0.25 | -0.53 ± 0.27 | -0.79 ± 0.28 | 0.416 | 0.832 | 0.525 |
| Ct.BMD [baseline-mapped] [%] | -1.28 ± 0.39 | -1.01 ± 0.39 | -0.61 ± 0.45 | 0.664 | 0.285 | 0.516 |
| Ct.TMD [%] | -0.46 ± 0.16 | -0.26 ± 0.17 | -0.46 ± 0.18 | 0.447 | 0.991 | 0.438 |
| Ct.Th [%] | -0.65 ± 0.30 | -0.14 ± 0.32 | -0.83 ± 0.33 | 0.307 | 0.707 | 0.158 |
| Ct.Ar [%] | -0.50 ± 0.26 | 0.51 ± 0.27 | -0.72 ± 0.28 | **0.024** | 0.582 | **0.006** |
| Ct.Po.V [standard] [%] | 12.03 ± 2.84 | 6.08 ± 2.98 | 2.92 ± 3.13 | 0.205 | *0.051* | 0.479 |
| Ct.Po.V [baseline-mapped] [%] | 15.47 ± 3.92 | 7.68 ± 3.95 | 4.27 ± 4.56 | 0.217 | *0.087* | 0.591 |
| Ct.Po [standard] [%] | 12.78 ± 2.78 | 5.49 ± 2.91 | 4.14 ± 3.06 | 0.116 | *0.058* | 0.756 |
| Ct.Po [baseline-mapped] [%] | 17.78 ± 4.35 | 9.79 ± 4.38 | 5.88 ± 5.06 | 0.252 | 0.100 | 0.579 |
| Ct.Po.Dm [standard] [%] | 0.78 ± 0.69 | 0.84 ± 0.72 | 0.42 ± 0.76 | 0.959 | 0.739 | 0.699 |
| Ct.Po.Dm [baseline-mapped] [%] | 1.15 ± 0.79 | -0.38 ± 0.79 | 1.03 ± 0.92 | 0.228 | 0.922 | 0.277 |
| Tb.N [%] | -0.93 ± 0.57 | -0.21 ± 0.60 | 0.53 ± 0.63 | 0.433 | 0.110 | 0.407 |
| Tb.Th [%] | 0.90 ± 0.59 | 0.53 ± 0.62 | -0.27 ± 0.65 | 0.699 | 0.212 | 0.388 |
| Tb.Sp [%] | 1.13 ± 0.62 | 0.28 ± 0.65 | -0.40 ± 0.68 | 0.402 | 0.125 | 0.482 |
| Tb.Sp.SD [%] | 1.89 ± 0.97 | 0.34 ± 1.02 | 0.01 ± 1.07 | 0.327 | 0.223 | 0.829 |
| ***Biomechanics*** |  |  |  |  |  |  |
| Stiffness, K [%] | -0.99 ± 0.63 | -0.53 ± 0.66 | -1.86 ± 0.70 | 0.654 | 0.379 | 0.186 |
| App Modulus, E [%] | -1.19 ± 0.65 | -0.82 ± 0.68 | -2.15 ± 0.71 | 0.726 | 0.345 | 0.198 |
| Estimated Failure Load, F [%] | -0.92 ± 0.56 | -0.47 ± 0.58 | -1.62 ± 0.61 | 0.614 | 0.426 | 0.195 |
| Ct.LF _dist_ [%] | 0.22 ± 0.71 | 0.92 ± 0.75 | 0.19 ± 0.79 | 0.548 | 0.973 | 0.512 |
| Ł adjusted for race and Δ BMI;  HR-pQCT = high resolution peripheral quantitative computed tomography; T2D = type 2 diabetes; BMD = bone mineral density; Tt.BMD = total bone mineral density; Tb.BMD = trabecular BMD; Ct.BMD = cortical BMD; Ct.TMD = cortical tissue mineral density; Ct.Th = cortical thickness; Ct.Ar = cortical area; Ct.Po.V = intracortical pore volume; Ct.Po = intracortical porosity; Ct.Po.Dm = mean cortical pore diameter; Tb.N = trabecular number; Tb.Th = trabecular thickness, Tb.Sp = trabecular separation; Tb.Sp.SD = standard deviation of intertrabecular distances; Ct.LF dist = distal cortical load fraction; App Modulus = apparent modulus;  [standard] = the standard analysis method was used to compute the respective parameter: the cortical region was identified independently in both the baseline and follow-up images on scans that were matched on total cross-sectional area.  [baseline-mapped] = baseline-mapped parameters were computed using a postprocessing method in which first a rigid transformation was applied that aligns the follow-up to the baseline scan and maps forward the baseline cortical border to the follow-up scan so that exactly the same region of the bone is measured as cortical bone, even if the bone may have undergone endocortical trabecularization during the FU-time. | | | | | | |

| **Supplemental Table 4. Adjusted, annualized absolute changes for HR-pQCT measured bone microarchitectural parameters at the ultradistal tibia.** Results are given for non-diabetic, postmenopausal female controls (Co), T2D postmenopausal women without history of fragility fractures (DM), and T2D postmenopausal women with a positive history of fragility fractures (DMFx). Shown are adjusted means with standard errors (SEM). Significant p-values (p<0.05) are marked in bold print, statistical trends are printed in italics. | | | | | | |
| --- | --- | --- | --- | --- | --- | --- |
|  | **Mean annualized absolute changes ^ł^ ± SEM** | | | **p- values** | | |
| **Ultradistal Tibia** | **Co (n=12)** | **DM (n=10)** | **DMFX (n=10)** | **Co vs.**  **DM** | **Co vs. DMFx** | **DM vs. DMFx** |
| ***Basic HR-pQCT measures*** |  |  |  |  |  |  |
| Tt.BMD [mg/cm^3^] | -1.86 ± 0.66 | -1.17 ± 0.64 | -2.53 ± 0.63 | 0.495 | 0.498 | 0.138 |
| Tb.BMD [mg/cm^3^] | 0.39 ± 0.33 | 0.46 ± 0.32 | 0.02 ± 0.31 | 0.895 | 0.452 | 0.333 |
| Ct.BMD [standard] [mg/cm^3^] | -9.35 ± 2.39 | -6.87 ± 2.34 | -10.78 ± 2.28 | 0.501 | 0.690 | 0.238 |
| Ct.BMD [baseline-mapped] [mg/cm^3^] | -13.14 ± 2.39 | -7.10 ± 2.34 | - 11.31 ± 2.29 | 0.109 | 0.610 | 0.203 |
| Ct.TMD [mg/cm^3^] | -5.40 ± 1.33 | -4.07 ± 1.29 | - 5.41 ± 1.26 | 0.516 | 0.997 | 0.463 |
| Ct.Th [µm] | -3.50 ± 3.68 | -0.27 ± 3.59 | -0.94 ± 3.51 | 0.569 | 0.643 | 0.894 |
| Ct.Ar [mm^2^] | -0.67 ± 0.32 | -0.25 ± 0.31 | -0.27 ± 0.30 | 0.393 | 0.394 | 0.978 |
| Ct.Po.V [standard] [mm^3^] | 3.20 ± 1.33 | 2.85 ± 1.30 | 5.09 ± 1.27 | 0.865 | 0.347 | 0.225 |
| Ct.Po.V [baseline-mapped] [mm^3^] | 4.12 ± 1.09 | 2.66 ± 1.07 | 5.04 ± 1.04 | 0.385 | 0.577 | 0.119 |
| Ct.Po [standard] [%] | 0.41 ± 0.13 | 0.32 ± 0.13 | 0.57 ± 0.13 | 0.651 | 0.451 | 0.188 |
| Ct.Po [baseline-mapped] [%] | 0.65 ± 0.14 | 0.34 ± 0.14 | 0.65 ± 0.13 | 0.152 | 0.977 | 0.116 |
| Ct.Po.Dm [standard] [µm] | 4.31 ± 1.13 | 1.16 ± 1.10 | 0.11 ± 0.11 | *0.079* | **0.019** | 0.499 |
| Ct.Po.Dm [baseline-mapped] [µm] | 5.79 ± 1.08 | 1.53 ± 1.05 | 0.97 ± 1.03 | **0.016** | **0.006** | 0.703 |
| Tb.N [mm^-1^] | -0.04 ± 0.01 | -0.02 ± 0.01 | 0.02 ± 0.01 | 0.192 | **0.004** | *0.061* |
| Tb.Th [µm] | 2.22 ± 0.64 | 1.14 ± 0.63 | -0.59 ± 0.61 | 0.285 | **0.007** | *0.056* |
| Tb.Sp [µm] | 11.77 ± 3.17 | 5.38 ± 3.10 | -4.11 ± 3.03 | 0.197 | **0.003** | **0.037** |
| Tb.Sp.SD [µm] | 4.79 ± 2.07 | 2.95 ± 2.02 | -3.40 ± 1.98 | 0.562 | **0.013** | **0.032** |
| ***Biomechanics*** |  |  |  |  |  |  |
| Stiffness, K [N/mm] | 149.09 ± 491.74 | -281.31 ± 480.38 | -1880.41 ± 470.15 | 0.569 | **0.010** | **0.024** |
| App Modulus, E [MPa] | 3.05 ± 5.25 | -2.40 ± 5.13 | -20.37 ± 5.02 | 0.501 | **0.006** | **0.018** |
| Estimated Failure Load, F [N] | -3.00 ± 20.94 | -15.74 ± 20.45 | -83.76 ± 20.02 | 0.692 | **0.016** | **0.024** |
| Ct.LF _dist_ [%] | -0.90 ± 0.19 | -0.37 ± 0.19 | -0.19 ± 0.18 | *0.076* | **0.019** | 0.507 |
| Ł adjusted for race and Δ BMI  HR-pQCT = high resolution peripheral quantitative computed tomography; T2D = type 2 diabetes; BMD = bone mineral density; Tt.BMD = total bone mineral density; Tb.BMD = trabecular BMD; Ct.BMD = cortical BMD; Ct.TMD = cortical tissue mineral density; Ct.Th = cortical thickness; Ct.Ar = cortical area; Ct.Po.V = intracortical pore volume; Ct.Po = intracortical porosity; Ct.Po.Dm = mean cortical pore diameter; Tb.N = trabecular number; Tb.Th = trabecular thickness, Tb.Sp = trabecular separation; Tb.Sp.SD = standard deviation of intertrabecular distances; Ct.LF dist = distal cortical load fraction; App Modulus = apparent modulus;  [standard] = the standard analysis method was used to compute the respective parameter: the cortical region was identified independently in both the baseline and follow-up images on scans that were matched on total cross-sectional area.  [baseline-mapped] = baseline-mapped parameters were computed using a postprocessing method in which first a rigid transformation was applied that aligns the follow-up to the baseline scan and maps forward the baseline cortical border to the follow-up scan so that exactly the same region of the bone is measured as cortical bone, even if the bone may have undergone endocortical trabecularization during the FU time. | | | | | | |

| **Supplemental Table 5. Adjusted, annualized absolute changes for HR-pQCT measured bone microarchitectural parameters at the ultradistal radius.** Results are given for non-diabetic, postmenopausal female controls (Co), T2D postmenopausal women without history of fragility fractures (DM), and T2D postmenopausal women with a positive history of fragility fractures (DMFx). Shown are adjusted means with standard errors (SEM). Significant p-values (p<0.05) are marked in bold print, statistical trends are printed in italics. | | | | | | |
| --- | --- | --- | --- | --- | --- | --- |
|  | **Mean annualized absolute changes ^ł^ ± SEM** | | | **p- values** | | |
| **Ultradistal Radius** | **Co (n=11)** | **DM (n=9)** | **DMFX (n=7)** | **Co vs.**  **DM** | **Co vs. DMFx** | **DM vs. DMFx** |
| ***Basic HR-pQCT measures*** |  |  |  |  |  |  |
| Tt.BMD [mg/cm^3^] | -2.52 ± 0.76 | 0.04 ± 0.80 | -3.16 ± 0.84 | *0.050* | 0.586 | **0.014** |
| Tb.BMD [mg/cm^3^] | -0.43 ± 0.43 | 0.36 ± 0.45 | 0.22 ± 0.47 | 0.263 | 0.339 | 0.831 |
| Ct.BMD [standard] [mg/cm^3^] | -8.08 ± 2.26 | -4.68 ± 2.37 | -6.88 ± 2.49 | 0.359 | 0.734 | 0.536 |
| Ct.BMD [baseline-mapped] [mg/cm^3^] | -12.02 ± 3.57 | -9.28 ± 3.59 | -5.60 ± 4.15 | 0.626 | 0.268 | 0.525 |
| Ct.TMD [mg/cm^3^] | -4.43 ± 1.55 | -2.56 ± 1.62 | -4.35 ± 1.71 | 0.456 | 0.973 | 0.460 |
| Ct.Th [µm] | 4.72 ± 2.49 | 1.03 ± 2.60 | 7.68 ± 2.74 | 0.363 | 0.447 | *0.098* |
| Ct.Ar [mm^2^] | -0.27 ± 0.14 | 0.28 ± 0.14 | -0.38 ± 0.15 | **0.021** | 0.610 | **0.006** |
| Ct.Po.V [standard] [mm^3^] | 0.95 ± 0.34 | 0.93 ± 0.36 | 0.62 ± 0.38 | 0.977 | 0.534 | 0.554 |
| Ct.Po.V [baseline-mapped] [mm^3^] | 1.06 ± 0.32 | 0.82 ± 0.32 | 0.63 ± 0.37 | 0.636 | 0.402 | 0.708 |
| Ct.Po [standard] [%] | 0.27 ± 0.08 | 0.19 ± 0.09 | 0.20 ± 0.09 | 0.553 | 0.569 | 0.963 |
| Ct.Po [baseline-mapped] [%] | 0.36 ± 0.10 | 0.28 ± 0.10 | 0.21 ± 0.12 | 0.626 | 0.383 | 0.692 |
| Ct.Po.Dm [standard] [µm] | 1.42 ± 1.27 | 1.28 ± 1.33 | 0.71 ± 1.40 | 0.946 | 0.720 | 0.773 |
| Ct.Po.Dm [baseline-mapped] [µm] | 2.14 ± 1.52 | -0.10 ± 1.52 | 1.81 ± 1.76 | 0.203 | 0.893 | 0.262 |
| Tb.N [mm^-1^] | -0.02 ± 0.01 | -0.003 ± 0.01 | 0.01 ± 0.01 | 0.474 | 0.146 | 0.458 |
| Tb.Th [µm] | 0.59 ± 0.39 | 0.30 ± 0.41 | -0.30 ± 0.43 | 0.648 | 0.151 | 0.323 |
| Tb.Sp [µm] | 5.85 ± 2.91 | 1.63 ± 3.04 | -1.91 ± 3.20 | 0.374 | *0.099* | 0.438 |
| Tb.Sp.SD [µm] | 4.86 ± 2.12 | 1.10 ± 2.22 | 0.80 ± 2.34 | 0.281 | 0.230 | 0.929 |
| ***Biomechanics*** |  |  |  |  |  |  |
| Stiffness, K [kN/mm] | -503.92 ± 305.16 | -172.83 ±  319.77 | -940.38 ± 335.91 | 0.504 | 0.364 | 0.119 |
| App Modulus, E [MPa] | -13.30 ± 7.27 | -10.01 ± 7.62 | -26.41 ± 8.00 | 0.779 | 0.255 | 0.159 |
| Estimated Failure Load, F [N] | -24.86 ± 14.12 | -9.33 ± 14.80 | -41.10 ± 15.55 | 0.498 | 0.463 | 0.160 |
| Ct.LF _dist_ [%] | 0.08 ± 0.32 | 0.47 ± 0.34 | 0.01 ± 0.36 | 0.463 | 0.895 | 0.375 |
| Ł adjusted for race and Δ BMI  HR-pQCT = high resolution peripheral quantitative computed tomography; T2D = type 2 diabetes; BMD = bone mineral density; Tt.BMD = total bone mineral density; Tb.BMD = trabecular BMD; Ct.BMD = cortical BMD; Ct.TMD = cortical tissue mineral density; Ct.Th = cortical thickness; Ct.Ar = cortical area; Ct.Po.V = intracortical pore volume; Ct.Po = intracortical porosity; Ct.Po.Dm = mean cortical pore diameter; Tb.N = trabecular number; Tb.Th = trabecular thickness, Tb.Sp = trabecular separation; Tb.Sp.SD = standard deviation of intertrabecular distances; Ct.LF dist = distal cortical load fraction; App Modulus = apparent modulus;  [standard] = the standard analysis method was used to compute the respective parameter: the cortical region was identified independently in both the baseline and follow-up images on scans that were matched on total cross-sectional area.  [baseline-mapped] = baseline-mapped parameters were computed using a postprocessing method in which first a rigid transformation was applied that aligns the follow-up to the baseline scan and maps forward the baseline cortical border to the follow-up scan so that exactly the same region of the bone is measured as cortical bone, even if the bone may have undergone endocortical trabecularization during the FU-time. | | | | | | |

| **Supplemental Table 6. Adjusted, annual percent changes (%) for HR-pQCT measured bone microarchitectural parameters at the ultradistal tibia.** Results are given for non-diabetic, postmenopausal female controls (Co), T2D postmenopausal women without history of fragility fractures (DM), and T2D postmenopausal women with a positive history of fragility fractures (DMFx). Shown are adjusted means with adjustments for age, race, and delta BMI with standard errors (SEM). Significant p-values (p<0.05) are marked in bold print, statistical trends are printed in italics. | | | | | | |
| --- | --- | --- | --- | --- | --- | --- |
|  | **Mean annual percent changes ^ł^ ± SEM** | | | **p- values** | | |
| **Ultradistal Tibia** | **Co (n=12)** | **DM (n=10)** | **DMFX (n=10)** | **Co vs.**  **DM** | **Co vs. DMFx** | **DM vs. DMFx** |
| ***Basic HR-pQCT measures*** |  |  |  |  |  |  |
| Tt.BMD [%] | -0.66 ± 0.21 | -0.34 ± 0.21 | -1.04 ± 0.21 | 0.326 | 0.238 | **0.028** |
| Tb.BMD [%] | 0.21 ± 0.15 | 0.38 ± 0.15 | -0.004 ± 0.15 | 0.475 | 0.377 | *0.097* |
| Ct.BMD [standard] [%] | -1.11 ± 0.29 | -0.75 ± 0.29 | -1.36 ± 0.29 | 0.419 | 0.580 | 0.154 |
| Ct.BMD [baseline-mapped] [%] | -1.58 ± 0.29 | -0.78 ± 0.29 | -1.41 ± 0.29 | *0.085* | 0.709 | 0.142 |
| Ct.TMD [%] | -0.56 ± 0.13 | -0.38 ± 0.13 | -0.63 ± 0.13 | 0.377 | 0.702 | 0.183 |
| Ct.Th [%] | -0.50 ± 0.33 | 0.002 ± 0.34 | -0.05 ± 0.33 | 0.340 | 0.386 | 0.917 |
| Ct.Ar [%] | -0.73 ± 0.30 | -0.19 ± 0.30 | -0.22 ± 0.29 | 0.242 | 0.267 | 0.939 |
| Ct.Po.V [standard] [%] | 3.58 ± 1.83 | 4.14 ± 1.82 | 7.67 ± 1.81 | 0.841 | 0.152 | 0.189 |
| Ct.Po.V [baseline-mapped] [%] | 6.52 ± 1.71 | 4.66 ± 1.79 | 8.12 ± 1.69 | 0.482 | 0.539 | 0.168 |
| Ct.Po [standard] [%] | 4.42 ± 1.95 | 4.39 ± 1.94 | 8.36 ± 1.93 | 0.994 | 0.195 | 0.168 |
| Ct.Po [baseline-mapped] [%] | 7.32 ± 1.83 | 5.26 ± 1.83 | 8.51 ± 1.82 | 0.468 | 0.669 | 0.225 |
| Ct.Po.Dm [standard] [%] | 2.12 ± 0.55 | 0.60 ± 0.55 | 0.10 ± 0.54 | *0.083* | **0.024** | 0.531 |
| Ct.Po.Dm [baseline-mapped] [%] | 2.82 ± 0.54 | 0.84 ± 0.54 | 0.50 ± 0.54 | **0.025** | **0.010** | 0.665 |
| Tb.N [%] | -2.22 ± 0.64 | -0.97 ± 0.63 | 1.03 ± 0.63 | 0.211 | **0.003** | **0.038** |
| Tb.Th [%] | 2.83 ± 0.82 | 1.54 ± 0.82 | -0.88 ± 0.81 | 0.312 | **0.007** | *0.050* |
| Tb.Sp [%] | 2.45 ± 0.70 | 1.10 ± 0.70 | -0.85 ± 0.69 | 0.216 | **0.005** | *0.063* |
| Tb.Sp.SD [%] | 2.39 ± 0.76 | 1.06 ± 0.76 | -0.89 ± 0.75 | 0.266 | **0.009** | *0.085* |
| ***Biomechanics*** |  |  |  |  |  |  |
| Stiffness, K [%] | 0.08 ± 0.32 | -0.09 ± 0.32 | -1.47 ± 0.32 | 0.721 | **0.004** | **0.006** |
| App Modulus, E [%] | 0.20 ± 0.32 | -0.08 ± 0.31 | -1.46 ± 0.31 | 0.564 | **0.002** | **0.006** |
| Estimated Failure Load, F [%] | -0.05 ± 0.27 | -0.15 ± 0.27 | -1.24 ± 0.26 | 0.820 | **0.007** | **0.009** |
| Ct.LF _dist_ [%] | -2.19 ± 0.41 | -0.88 ± 0.41 | -0.19 ± 0.41 | **0.049** | **0.004** | 0.253 |
| Ł adjusted for age, race and Δ BMI  HR-pQCT = high resolution peripheral quantitative computed tomography; T2D = type 2 diabetes; BMD = bone mineral density; Tt.BMD = total bone mineral density; Tb.BMD = trabecular BMD; Ct.BMD = cortical BMD; Ct.TMD = cortical tissue mineral density; Ct.Th = cortical thickness; Ct.Ar = cortical area; Ct.Po.V = intracortical pore volume; Ct.Po = intracortical porosity; Ct.Po.Dm = mean cortical pore diameter; Tb.N = trabecular number; Tb.Th = trabecular thickness, Tb.Sp = trabecular separation; Tb.Sp.SD = standard deviation of intertrabecular distances; Ct.LF dist = distal cortical load fraction; App Modulus = apparent modulus;  [standard] = the standard analysis method was used to compute the respective parameter: the cortical region was identified independently in both the baseline and follow-up images on scans that were matched on total cross-sectional area.  [baseline-mapped] = baseline-mapped parameters were computed using a postprocessing method in which first a rigid transformation was applied that aligns the follow-up to the baseline scan and maps forward the baseline cortical border to the follow-up scan so that exactly the same region of the bone is measured as cortical bone, even if the bone may have undergone endocortical trabecularization during the FU time. | | | | | | |
